# Arthroscopic meniscal surgery in Norway from 2010 to 2020: A paradigmatic shift

**DOI:** 10.1002/jeo2.70113

**Published:** 2024-12-12

**Authors:** Karoline Nysted Nilsen, Frank‐David Øhrn, Asbjørn Årøen, Tor Åge Myklebust, Tommy Frøseth Aae

**Affiliations:** ^1^ Department of Orthopedic Surgery St. Olavs Hospital Trondheim Norway; ^2^ Department of Orthopedic Surgery, Kristiansund Hospital Møre and Romsdal Hospital Trust Kristiansund Norway; ^3^ Department of Neuromedicine and Movement Science, Faculty of Medicine and Health Sciences NTNU Trondheim Norway; ^4^ Department of Orthopedic Surgery Akershus University Hospital Nordbyhagen Norway; ^5^ Institute of Clinical Medicine (Campus AHUS), Faculty of Medicine University of Oslo Oslo Norway; ^6^ Department of Registration The Cancer Registry of Norway Oslo Norway; ^7^ Department of Research and Innovation Møre and Romsdal Hospital Trust Kristiansund Norway

**Keywords:** arthroscopy, incidence, meniscal injury, meniscal repair, meniscal resection

## Abstract

**Purpose:**

Meniscal injuries in the knee are usually treated surgically with arthroscopic partial resection (APR) or arthroscopic repair (AR). APR has been shown to increase the risk of osteoarthritis and the focus has shifted to repairing the meniscus with AR. The extent of this shift is yet to be established and an analysis of incidence rates (IR) of APR and AR for meniscal injuries could highlight this.

**Methods:**

Data from the Norwegian Patient Registry (NPR) and Statistics Norway (SN) from 2010 to 2020 were collected. The number of procedures, demographics and facilities providing meniscal surgery were obtained from NPR, while population size and catchment area were collected from SN. IR of APR and AR and APR/AR rate ratios were estimated and compared.

**Results:**

A total of 119,528 knee arthroscopies were performed, 89.6% of which were APR. The number of APR performed nationally decreased by 72%, while AR procedures increased by 178%. The national IR of APR decreased from 298 to 82/100,000 inhabitants (*p* < 0.001). For AR, the national IR increased annually from 13/100,000 inhabitants to a peak in 2019 of 32/100,000 inhabitants (*p* < 0.001). The APR/AR rate ratio decreased from 22 to below five and the APR/AR trend curves showed a statistically significant decrease (*p* < 0.001).

**Conclusion:**

Surgical treatment of meniscal injuries has changed, with a substantial reduction in APR and a strong increase in AR. The reduction in APR, especially in older patients, suggests that meniscal surgery in Norway has undergone a paradigmatic shift, in line with recent literature.

**Level of Evidence:**

Level IV.

**Abbreviations**
APR: arthroscopic partial resection
AR: arthroscopic repair
IR: incidence rate
IRR: incidence rate ratio
NPR: Norwegian Patient Registry
RHT: Regional Health Trust
SN: Statistics Norway

## INTRODUCTION

Meniscal injuries are among the most prevalent intra‐articular knee injuries and can be classified according to aetiology [9]. The traumatic injury occurs in young, active individuals, often with an acute onset, whereas the degenerative injury in middle‐aged and elderly individuals arises gradually [9]. Meniscal injuries are one of the most frequent causes of orthopaedic surgical procedures and are usually treated surgically with arthroscopic partial resection (APR) or arthroscopic repair (AR) [9, 10]. The AR procedure is associated with a long rehabilitation schedule and uncertainty as to whether the repair will heal [4, 22]. APR has a shorter recovery time after surgery, and most patients can return to sport within a few weeks or months.

Even though the short‐term effects of APR seem beneficial, APR has been shown to predispose to osteoarthritis and the symptomatic effect of APR for treating meniscal injuries is not as good as first assumed [7, 19, 26]. Notwithstanding increasing evidence that APR lacks added benefits over nonoperative treatments for degenerative injuries, several studies have demonstrated an increase in APR procedures in the 2010s [2, 28]. Recently, this topic has been highlighted by the orthopaedic community through initiatives that have resulted in a consensus that meniscal injuries should preferably be repaired or left alone [16, 23]. Despite this, reports have stated that the implementation of high‐level evidence into the practice of arthroscopic surgery is delayed [28]. For this reason, it is yet to be established whether preserving the meniscus has taken place in clinical practice with a decrease in APR and an increase in AR in meniscal procedures.

The purpose of this study was to calculate and compare incidence rates (IR) of APR and AR over a 10‐year period in Norway and to determine whether there are age, gender and regional effects on the IR of APR and AR. We hypothesised that the IR of APR would decrease from 2010 to 2020.

## MATERIALS AND METHODS

A cross‐sectional study was conducted in Norway in late 2023. All patients of any age who underwent APR or AR in Norway from 2010 to 2020 were included.

Statistics Norway (SN) is an independent professional institution in Norway responsible for collecting and publishing official statistics on the population at national, regional and local level. Data have been collected and published since 1971 and are publicly available. The data retrieved from SN included annual population size at the national level and annual catchment areas at the regional level based on Norway's four regional health trusts (RHTs) (Southern‐Eastern Norway [SEN]; Western Norway; Central Norway [CN] and Northern Norway [NN]) in the study period from 2010 to 2020.

The Norwegian Patient Registry (NPR) is a nationwide health registry established in 1997. The main function of the NPR is to collect information about patients who are undergoing or awaiting treatment in public or private specialist healthcare in Norway. To identify relevant patients in the NPR database, an electronic search was performed based on predefined procedure codes using the Nordic Medico‐Statistical Committee Classification of Surgical Procedures (NGD 11—Arthroscopic partial excision of meniscus of knee and NGD 21—Arthroscopic reinsertion of meniscus of knee) [21]. The NPR data contained the numbers of APRs and ARs performed by each of the RHTs or private institutions each year and in total during the study period, and patients were categorised by age groups (0–19, 20–39, 40–59 and 60+) and gender.

The IRs of APR and AR were calculated and the rate ratio between the two procedures, the incidence rate ratio (IRR), was calculated with point estimates and associated 95% confidence intervals. Linear trends in the rates for each group were tested by fitting Poisson regression models with year as a continuous covariate. IRs per year for each RHT and for the national average were compared using Wald tests after fitting Poisson regression models for each outcome including year (continuous) and RHT as covariates, as well as the corresponding interaction terms. A *p *< 0.05 deemed statistically significant. The data were analysed using Stata version 18.0.

## RESULTS

From 2010 to 2020, the population of Norway increased by 0.5 million, reaching 5.3 million at the end of 2020, with the SEN RHT catchment area being the most populous (Table 1). During the study period, a total of 119,528 knee arthroscopies for meniscal injuries were performed, of which APR accounted for 107,117 (89.6%) (Table 2). Of all APRs performed, public hospitals performed 66.4%, while private institutions accounted for 33.6% (71,182 and 35,935, respectively). For AR, public hospitals accounted for 89.3% (11,091), whereas the private healthcare sector performed 10.7% (1320). Over the study period, the annual number of APRs performed nationally decreased, while ARs increased (Table 2). These trends of a decrease in APR and an increase in AR were similar for both public RHTs and private institutions.

**Table 1:** The population of Norway from 2010 to 2020.

Year	National	SEN	WN	CN	NN
2010	4,858,199	2,707,012	1,012,202	673,364	465,621
2011	4,920,305	2,743,875	1,028,069	680,110	468,251
2012	4,985,870	2,785,259	1,041,886	687,968	470,757
2013	5,051,275	2,821,116	1,058,994	696,602	474,563
2014	5,109,056	2,854,217	1,073,836	702,869	478,134
2015	5,165,802	2,888,729	1,086,829	709,504	480,740
2016	5,213,985	2,920,730	1,096,202	715,059	481,994
2017	5,258,317	2,950,547	1,102,253	720,870	484,647
2018	5,258,317	2,977,723	1,106,295	725,600	486,001
2019	5,328,212	3,002,385	1,109,923	729,452	486,452
2020	5,367,580	3,032,671	1,116,423	733,940	484,546

Abbreviations: CN, Central Norway; NN, Northern Norway; SEN, Southern‐Eastern Norway; WN, Western Norway.

**Table 2:** Distribution of arthroscopic meniscal procedures in Norway from 2010 to 2020.

Year	National		SEN		WN		CN		NN		Private	
	APR	AR	APR	AR	APR	AR	APR	AR	APR	AR	APR	AR
2010	14,474	615	5834	333	1691	108	1497	92	1691	22	4583	60
2011	13,349	612	6014	326	1672	119	1623	90	1672	33	3297	44
2012	12,663	671	6108	397	1616	125	1434	99	1616	50	2796	0
2013	14,973	780	5210	414	1744	155	1369	109	1744	49	5916	53
2014	13,158	1003	4163	510	1547	199	1244	83	1547	38	5576	173
2015	9562	1095	3019	606	1316	168	913	114	1316	35	3796	172
2016	7807	1236	2517	727	1227	210	677	114	1227	29	2935	156
2017	6593	1413	2022	834	992	261	555	129	992	29	2708	160
2018	5287	1557	1910	986	894	310	519	115	894	28	1654	118
2019	4828	1720	1801	1053	866	324	523	151	866	39	1378	153
2020	4423	1709	1572	921	819	341	493	163	819	53	1296	231

Abbreviations: APR, arthroscopic partial resection; AR, arthroscopic repair; CN, Central Norway; NN, Northern Norway; SEN, Southern‐Eastern Norway; WN, Western Norway.

**Figure 1:**
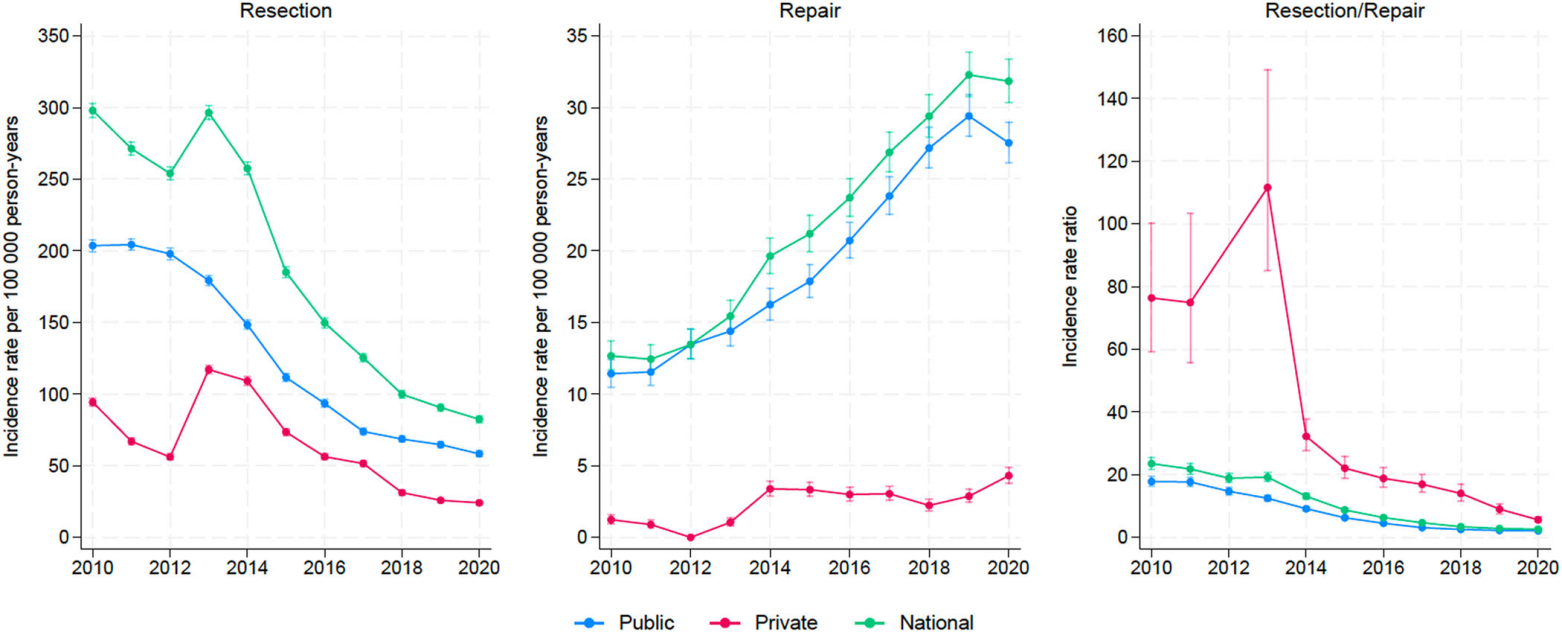
Incidence rates of meniscal procedures in Norway from 2010 to 2020.

The national IR of APR decreased significantly from 298/100,000 person‐years to 82/100,000 person‐years (*p *< 0.001) (Figure 1). For AR, the national IR increased significantly yearly from 13/100,000 person‐years to 32/100,000 person‐years (Figure 1). The APR/AR IRR decreased from 22 to under five at the national level, which was almost identical to the trend for public healthcare facilities (Figure 1). By contrast, the APR/AR IRR for private institutions increased from 75 in 2010 to 110 in 2013. From 2013 to 2014, a steep decline in this IRR occurred, and in 2020, the IRR for private institutions ended up at almost the national and public levels (Figure 1). The APR/AR trend curves all showed a statistically significant decrease in IRR (*p* < 0.001).

**Figure 2:**
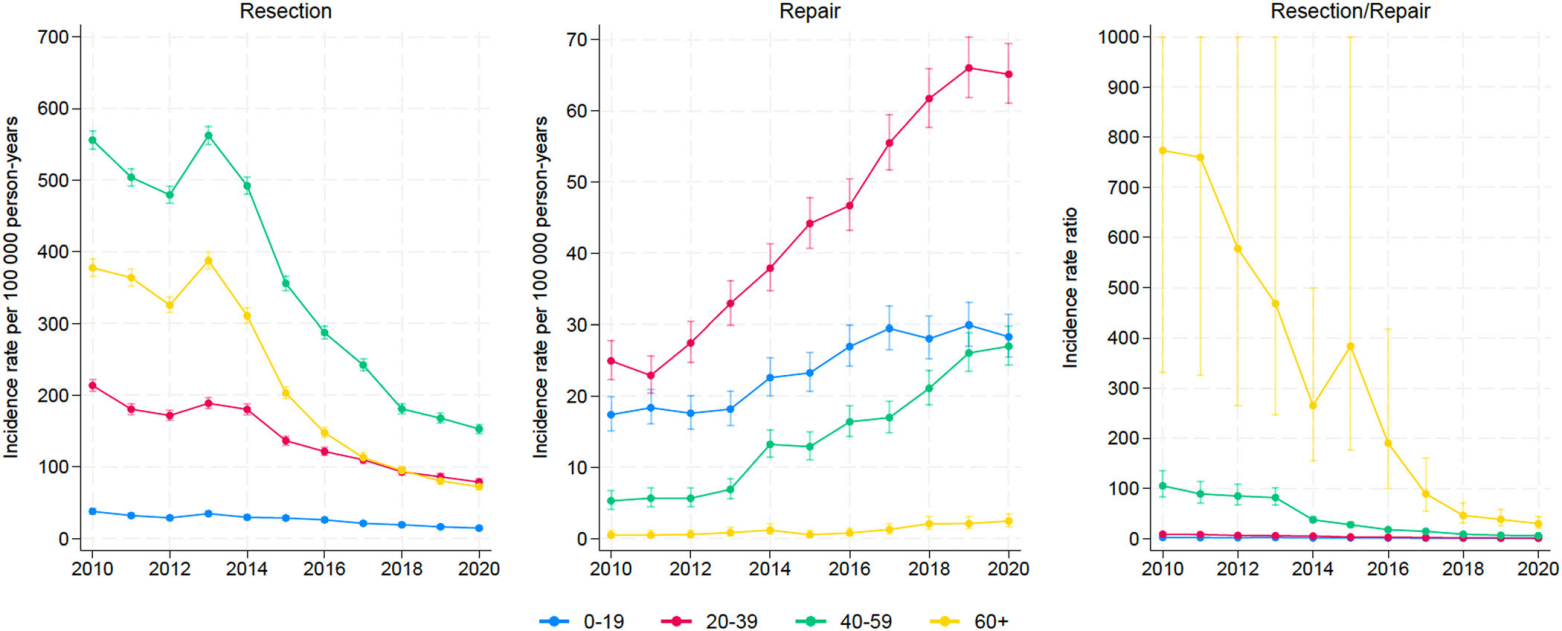
Incidence rates of meniscal procedures in Norway from 2010 to 2020 by age groups.

For all age groups, the national IR of APR decreased and the IR of AR increased significantly (*p* < 0.001) (Figure 2). The increase in AR was especially marked for patients between 20 and 59 years of age. The APR/AR trend curves were unchanged for patients younger than 40 years of age, whereas for older patients, they showed a statistically significant decrease in IRR, most notably in the oldest patients (*p* < 0.001) (Figure 2).

**Figure 3:**
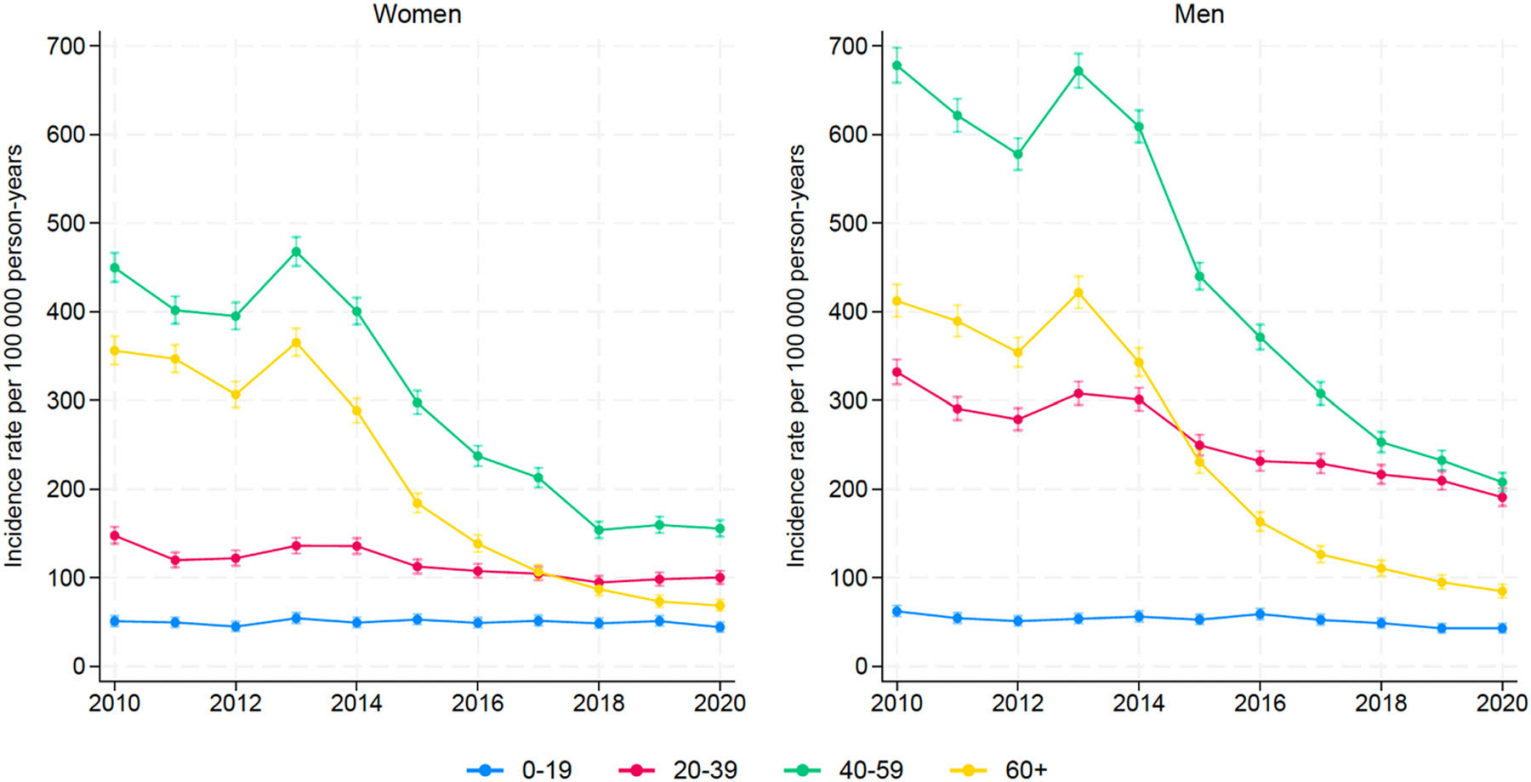
Incidence rates of meniscal procedures in Norway from 2010 to 2020 by gender.

Concerning gender, there was a higher incidence of interventions for males (*p* < 0.001) and the male age group of 40–59 years underwent the most meniscal surgeries (Figure 3). Gender did not discriminate procedures, and APR and AR were, therefore, treated as one procedure. For both genders, the IR decreased significantly during the study period and there was no statistical difference between genders. Trend curves showed a statistical difference for all male and female age groups (*p* < 0.001), except for the youngest women (*p* = 0.45).

**Figure 4:**
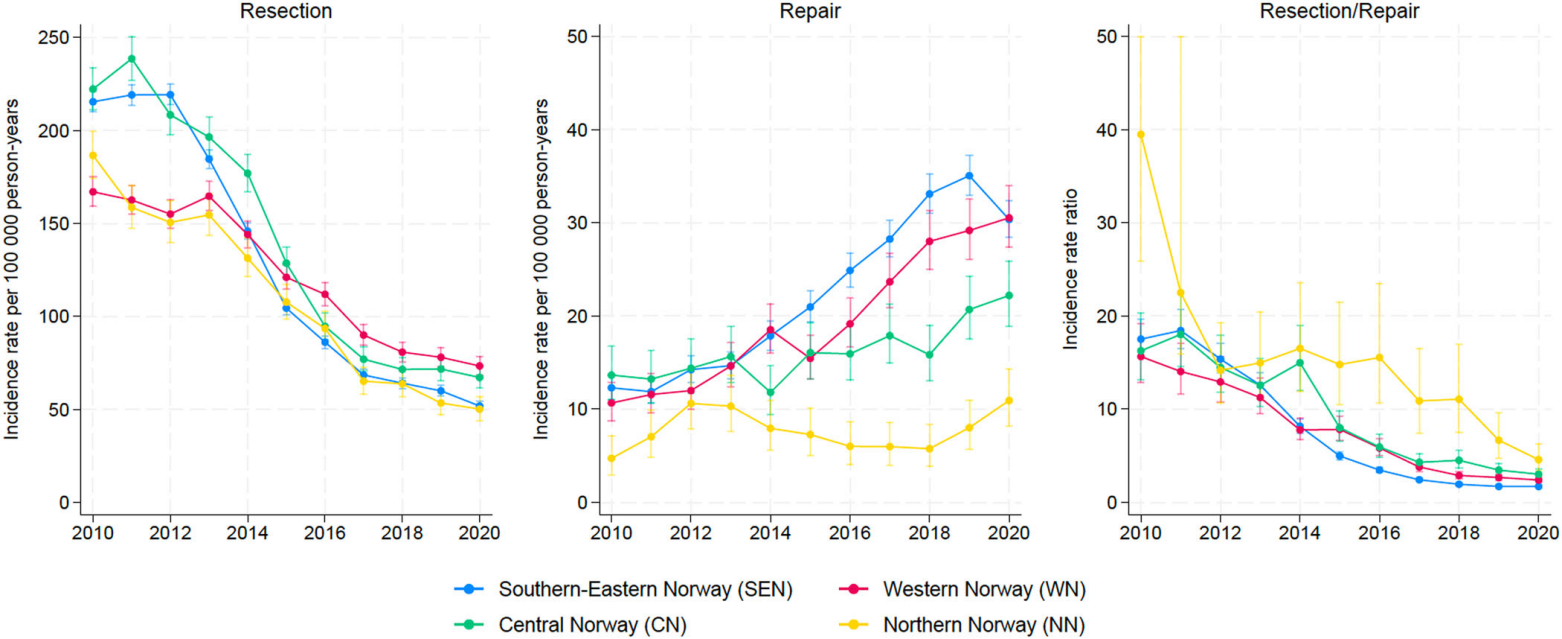
Incidence rates of meniscal procedures in regional health trusts in Norway from 2010 to 2020.

Public institutions made approximately twice as many APR as private institutions (*p* < 0.001), whereas for AR, public institutions accounted for nine times as many procedures (*p* < 0.001) (Figure 4). Both public and private institutions had a significant decrease in IR of APR and increase in AR (*p* < 0.001), although NN failed to demonstrate a statistically significant increase of AR (*p* = 0.47) (Figure 4). All regions except CN showed a significant decrease in APR, whereas all regions showed a significant increase in AR when testing for differences between regional‐specific IRRs and the national average IRR (Table 3).

**Table 3:** IRR per year for each regional health trust and for the national average.

	Resection			Repair		
	IRR/year	95% CI	*p* Value*	IRR/year	95% CI	*p* Value*
SEN	0.848	0.845–0.851	<0.001	1.122	1.113–1.131	<0.001
WN	0.912	0.907–0.917	<0.001	1.120	1.105–1.135	0.004
CN	0.864	0.858–0.869	0.463	1.050	1.032–1.069	<0.001
NN	0.874	0.866–0.881	0.026	1.011	0.981–1.043	<0.001
National	0.866	0.864–0.868		1.100	1.094–1.108	

Abbreviations: 95% CI, 95% confidence interval; CN, Central Norway; IRR, incidence rate ratio; NN, Northern Norway; SEN, Southern‐Eastern Norway; WN, Western Norway.

* Test for difference between region‐specific IRR and country‐average IRR.

## DISCUSSION

The most important finding of the present study was the significant decrease in APR during the study period. From 2010 to 2020, the number of APRs at the national level declined by almost three‐quarters from 14,474 to 4434, and the national IR for APR decreased by 72%. As for AR, our data demonstrated a significant threefold increase at the national level, and the IR of AR increased from 13 to 32 per 100,000 inhabitants. The detected change in IR for AR at the national level was not as marked as for APR and occurred more gradually.

Today, there is increased attention to meniscal injuries, as the trend in our society for all age groups is to remain active, which necessitates normal knee function [25]. Despite the fact that reports have demonstrated the disadvantages of APR [5, 19, 20, 27, 30], studies in the past decade have demonstrated an increase in APR [2, 3, 28]. Our results reveal that a change of scenario occurred in 2013, as APR in especially older patients decreased substantially. These patients mainly have degenerative meniscal injuries, which have been under scrutiny, as recent studies have demonstrated that surgical outcomes after APR are not superior to sham surgery or conservative measures [14, 26], while the associated costs are high and the conversion to knee arthroplasty is more common than following AR [17, 24].

As a consequence, conservative treatment including physiotherapy has been hailed as the first‐line treatment for degenerative meniscal injuries [1, 14, 15, 26, 29]. This change has also been emphasised by the orthopaedic community, resulting in a consensus on degenerative meniscal injuries that contributes to nonoperative management of these injuries [5, 6]. Together, a tendency toward an important shift in meniscal surgery can be seen, a massive but required reduction in APR that is correlated with nonoperative treatment of degenerative meniscal injuries that is consistent with reports from other countries [13, 17].

With regard to AR, patients aged 20–39 years had the largest increase in the IR of AR. For this population, most meniscal injuries are traumatic and not degenerative. The traumatic meniscal injuries are more seldom treated with nonoperative measures than degenerative injuries, and the increase in AR can be seen in light of recent clinical trials and consensus reports stating that traumatic meniscal injuries should be repaired and not resected [16, 20, 23, 30]. For the youngest patients, 0–19 years, only small changes in IR for both APR and AR were identified. One possible explanation for this finding is that arthroscopic surgery is rarely performed in this age group, and few surgeries took place during the study period.

Meniscal procedures are most frequently carried out in men [28], as also seen in our findings. Ye, our data reveal that the IR of meniscal procedures for all age groups for both men and women declined, with the exception of the youngest women. In men and women older than 60 years, there was a significant reduction in arthroscopic meniscal surgery of almost 75%. On the positive side, however, trend curves for both genders did not show differences in meniscal procedures, and men and women were treated in equal numbers.

The IR for meniscal procedures not only demonstrates variation with regard to age but also institution and region. A registry study from Denmark revealed that in particular, private institutions showed a considerable increase in the use of meniscal procedures [12]. Our study reveals that both public and private institutions showed a significant decrease in IR of APR, while the change was most notable in public institutions, as the IR of APR was decreased by almost 75%. The studies are not directly comparable as our study is based on data a decade later, but in the overlapping years of the study periods, the IR of APR are comparable.

Birkmeyer et al. reported that surgical variation was due to differences in surgeons' beliefs about the indications for surgery and whether patients' preferences were integrated into treatment decisions [8]. Even though NN showed a less marked increase in the IR of AR than the other RHTs, the reason might be multifactorial. For many years, NN has faced the challenges of an ageing population, emigration of the younger generations and low birth rates. This means that there are a large number of older patients with degenerative meniscal tears, where first‐line treatment is conservative measures, including physiotherapy. Strategies to reduce undesirable variation in the use of surgery have been described by McCulloch et al. [18]. In order to reduce regional variation, it is of paramount interest for healthcare personnel and patients that better scientific proof about the effectiveness of meniscal surgery is sought out and communicated.

Our study has certain limitations. The available data did not discriminate between degenerative and traumatic meniscal injuries, nor did they include classification of the meniscal injuries or the presence of newer meniscal injuries such as root tears and ramp lesions. When discussing degenerative and traumatic meniscal injuries, it was done in light of the patients' age, as younger patients are susceptible to traumatic injuries and older patients are susceptible to degenerative injuries. As for the comparison of genders, the data did not separate them for APR and AR. Nonetheless, when combining APR and AR, we could not detect any gender differences in the changes of IRs. Responsiveness to NPR is assumed to be close to 100% [11]. However, there might have been a few surgeries that were not reported to NPR and thus not included in our data, but we do not believe that this would have affected our findings. Finally, we have assumed that patients were treated in their own health trust, but some might have received surgery in another health region.

The major strength of this study is the accuracy of our results, as they are based on reliable national registries with responsiveness of up to 100%. Another important feature is that we have accounted for the whole population and assessed variations based on age, gender and region. Further, our study utilises recent data and is the first to report on meniscal trends up to 2020. The trend identified in our study is a warranted change and will hopefully reduce the incidence of degenerative osteoarthritis caused by the loss of the meniscus. Our findings demonstrate compliance with recent reports and initiatives on the treatment of meniscal injuries.

## CONCLUSION

Surgical treatment of meniscal injuries has changed in recent years, with a substantial reduction in APR and a major increase in AR. The reduction in APR, especially in older patients, suggests that meniscal surgery in Norway has undergone a paradigmatic shift, in line with recent literature.

## AUTHOR CONTRIBUTIONS

Karoline Nysted Nilsen performed the literature search and drafted and edited the article. Frank‐David Øhrn and Asbjørn Årøen co‐drafted and co‐edited the article and critically reviewed the manuscript. Tor Åge Myklebust co‐drafted and co‐edited the method, results and discussion sections and critically reviewed the manuscript. Tommy Frøseth Aae established the aim of the study and study design, co‐drafted and co‐edited the article and critically reviewed the manuscript. All authors made contributions to the design, were involved in the drafting and read and approved the final manuscript.

## CONFLICT OF INTEREST STATEMENT

The authors declare no conflicts of interest.

## ETHICS STATEMENT

As all data were based on already anonymous records, the Regional Ethics Committee deemed approval to be unnecessary. The study was approved by the Data Access Committee of Møre and Romsdal Hospital Trust (reference 2024/1184).

## Data Availability

The data that support the findings of this study are available from the Norwegian Patient Registry (NPR) and Statistics Norway (SN). Restrictions apply to the availability of these data, which were used under license for this study. Data are available from the authors with the permission of NPR and SN.
